# Severe varicella-zoster virus pneumonia: a multicenter cohort study

**DOI:** 10.1186/s13054-017-1731-0

**Published:** 2017-06-07

**Authors:** Adrien Mirouse, Philippe Vignon, Prescillia Piron, René Robert, Laurent Papazian, Guillaume Géri, Pascal Blanc, Christophe Guitton, Claude Guérin, Naïke Bigé, Antoine Rabbat, Aurélie Lefebvre, Keyvan Razazi, Muriel Fartoukh, Eric Mariotte, Lila Bouadma, Jean-Damien Ricard, Amélie Seguin, Bertrand Souweine, Anne-Sophie Moreau, Stanislas Faguer, Arnaud Mari, Julien Mayaux, Francis Schneider, Annabelle Stoclin, Pierre Perez, Julien Maizel, Charles Lafon, Frédérique Ganster, Laurent Argaud, Christophe Girault, François Barbier, Lucien Lecuyer, Jérôme Lambert, Emmanuel Canet

**Affiliations:** 1 Service de réanimation médicale, Hôpital Saint-Louis, AP-HP, Paris, France; 2 Service de réanimation polyvalente, CHU Limoges, Limoges, France; 3 CIC1435 CHU Limoges, Limoges, France; 4 INSERM U1092, Limoges, France; 5 Département de biostatistiques, Hôpital Saint-Louis, AP-HP, Paris, France; 6 Service de réanimation médicale, CHU de Poitiers, Poitiers, France; 7 Service de réanimation des détresses respiratoires et infections sévères, Hôpital Nord, AP-HM, Marseille, France; 8 Service de réanimation médicale, Hôpital Cochin, AP-HP, Paris, France; 9 Service de réanimation médico-chirurgicale, CH de Pontoise, Pontoise, France; 10 Service de réanimation médicale, CHU Hôtel Dieu, Nantes, France; 11 Réanimation médicale, Groupement hospitalier nord, Hospices civiles de Lyon, Université de Lyon, INSERM 955, Lyon, France; 12 Service de réanimation médicale, hôpital Saint-Antoine, AP-HP, Paris, France; 13 Service de réanimation pneumologique, Hôpital Cochin, AP-HP, Paris, France; 14 Service de réanimation médicale, Hôpital Henri Mondor, AP-HP, Créteil, France; 15 Service de réanimation médico-chirurgicale, Hôpital Tenon, AP-HP, Paris, France; 16 Service de réanimation médicale, Hôpital Bichat, AP-HP, Paris, France; 17 Service de Réanimation Médico-Chirurgicale, Hôpital Louis Mourier, F-92700 Colombes, France; 18 Université Paris Diderot, IAME, UMR 1137, Sorbonne Paris Cité, INSERM, AP-HP, F-75018 Paris, France; 19 Service de réanimation médicale, CHU de Caen, Caen, France; 20 Service de réanimation médicale, CHU Gabriel-Montpied, Clermont-Ferrand, France; 21 Service de réanimation polyvalente, CHRU de Lille - Hôpital Roger Salengro, Lille, France; 22 Département de Néphrologie et Transplantation d’organes, CHU de Toulouse, Toulouse, France; 23 Service de Réanimation Hôpital Purpan, Centre Hospitalier Universitaire de Toulouse, Toulouse, France; 24 Service de pneumologie et réanimation médicale, Hôpital Pitié-Salpêtrière, AP-HP, Paris, France; 25 Service de Réanimation Médicale, Hôpital de Hautepierre, U1121 et FMTS, Université de Strasbourg, Strasbourg, France; 26 Service de réanimation et surveillance continue, Institut Gustave-Roussy, Villejuif, France; 27 Service de réanimation médicale, hôpital Brabois, Nancy, France; 28 Service de réanimation médicale, CHU de Picardie, Amiens, France; 29 Service de réanimation médico-chirurgicale, hôpital d’Angoulême, Angoulême, France; 30 Service de réanimation médicale, hôpital E. Muller, Mulhouse, France; 31 Service de réanimation médicale, Hôpital E. Herriot, Hospices Civils de Lyon, Lyon, France; 32 Medical Intensive Care Unit, Rouen University Hospital, Rouen, France; 33 UPRES EA 3830-IRIB, Institute for Biomedical Research, Rouen University, Rouen, France; 34 Service de réanimation médicale, hôpital La Source, Orléans, France; 35 Service de réanimation polyvalente, CH Sud Francilien, Corbeil-Essonnes, France

**Keywords:** Varicella-Zoster virus, Pneumonia, Steroids, Mechanical ventilation, Intensive care unit

## Abstract

**Background:**

Pneumonia is a dreaded complication of varicella-zoster virus (VZV) infection in adults; however, the data are limited. Our objective was to investigate the clinical features, management, and outcomes of critically ill patients with VZV-related community-acquired pneumonia (VZV-CAP).

**Methods:**

This was an observational study of patients with VZV-CAP admitted to 29 intensive care units (ICUs) from January 1996 to January 2015.

**Results:**

One hundred and two patients with VZV-CAP were included. Patients were young (age 39 years (interquartile range 32–51)) and 53 (52%) were immunocompromised. Time since respiratory symptom onset was 2 (1–3) days. There was a seasonal distribution of the disease, with more cases during spring and winter time. All but four patients presented with typical skin rash on ICU admission. Half the patients received mechanical ventilation within 1 (1–2) day following ICU admission (the ratio of arterial oxygen partial pressure to fractional inspired oxygen (PaO_2_/FiO_2_) = 150 (80–284), 80% with acute respiratory distress syndrome (ARDS)). Sequential Organ Failure Assessment (SOFA) score on day 1 (odds ratio (OR) 1.90 (1.33–2.70); *p* < 0.001), oxygen flow at ICU admission (OR 1.25 (1.08–1.45); *p* = 0.004), and early bacterial co-infection (OR 14.94 (2.00–111.8); *p* = 0.009) were independently associated with the need for mechanical ventilation. Duration of mechanical ventilation was 14 (7–21) days. ICU and hospital mortality rates were 17% and 24%, respectively. All patients were treated with aciclovir and 10 received adjunctive therapy with steroids. Compared to 60 matched steroid-free controls, patients treated with steroids had a longer mechanical ventilation duration, ICU length of stay, and a similar hospital mortality, but experienced more ICU-acquired infections.

**Conclusions:**

Severe VZV-CAP is responsible for an acute pulmonary involvement associated with a significant morbidity and mortality. Steroid therapy did not influence mortality, but increased the risk of superinfection.

**Electronic supplementary material:**

The online version of this article (doi:10.1186/s13054-017-1731-0) contains supplementary material, which is available to authorized users.

## Background

Pneumonia is associated with significant morbidity and mortality worldwide [1]. The importance of viruses as pathogens responsible for community-acquired pneumonia (CAP) has been emphasized by several epidemic outbreaks over the last decade: severe acute respiratory syndrome (SARS), avian influenza A (H5N1) virus, and the 2009 pandemic influenza A (H1N1) [2–5]. It is estimated that about 200 million cases of viral CAP occur annually, accounting for 17 to 39% of CAP [6–10]. In the intensive care unit (ICU) setting, viruses have been reported to account for up to one-third of patients with severe CAP, with a similar mortality to that observed with bacterial pneumonia [11]. The availability of new molecular techniques (such as polymerase chain reaction (PCR)) has greatly increased our ability to detect a wide range of viruses in respiratory secretions [12–15]. Nevertheless, although convincing data are available for agents such as influenza virus or respiratory syncytial virus and their role in severe pneumonia, the role of other recently discovered viruses (bocavirus, coronavirus NL63) remains debated and requires further research [16–19].

Varicella-zoster virus (VZV) is one of eight herpes viruses known to cause human infection and is distributed worldwide. Varicella or chickenpox is defined as the VZV primo-infection. In adults, VZV infection is associated with a greater number of complications, of which pneumonia is the most common and serious. Since its initial description in 1942, VZV-related community-acquired pneumonia (VZV-CAP) has become increasingly recognized as a serious and potentially lethal complication of what was once considered a relatively benign and self-limited viral infection [20]. A study with systematic chest X-ray showed an incidence of 16.3% of VZV-CAP in adult patients [21]. Autopsy findings have demonstrated the role of VZV in fatal cases of pneumonia. Microscopic findings included pulmonary edema, extensive alveolar hemorrhage, and mononuclear cell infiltration with histological evidence of intranuclear inclusion bodies [22, 23].

Data on critically ill adult patients with VZV-CAP are limited to case reports and small case series. The purpose of this study was to describe the clinical, biological, and radiological features and the outcome of severe forms of VZV-CAP (i.e., requiring ICU admission) in a large cohort and to report its implication for ICU management.

## Methods

This retrospective study was conducted in 29 French adult ICUs (see Additional file 1: Table S1). This study has been approved by the French Intensive Care Society Ethics Committee (n°15-12) and an authorization to use patient data from the French Data Protection Agency (n°1868870). According to French law, a waiver of informed consent was obtained.

### Inclusion criteria

All adult patients (≥18 years old) admitted for VZV-CAP in the participating ICUs between 1 January 1996 and 1 January 2015 were included. Patients were identified from the ICU databases using codes J171 and B012 from the International Classification of Diseases system (ICD-10) system. All the medical records of patients were reviewed by two investigators (AM and EC).

### Data collection

The data reported in Tables 1, 2, and 3 were abstracted from medical charts. Chickenpox features at ICU admission were collected including skin rash, pulmonary involvement, neurological impairment, or abdominal symptoms. Patients were defined as immunosuppressed if they met one of the following criteria: hematopoietic stem cell or solid organ transplantation, hematological malignancy, solid tumor progressing or in remission less than 5 years, steroid treatment for more than 3 months, and other immunosuppressive drugs. Physiological variables, laboratory data, and radiographic findings (chest X-ray and computed tomography (CT) when available) on ICU admission were also reported. Thrombocytopenia was defined as platelet count <150 G/l and hepatitis as alanine aminotransferase and/or aspartate aminotransferase ≥3 N. Disease severity was assessed using the Sequential Organ Failure Assessment (SOFA) on day 1 after ICU admission [24]. Patients were classified as having acute respiratory failure (ARF) if they met the following criteria: severe dyspnea at rest; a respiratory rate greater than 30 breaths/min or clinical signs of respiratory distress; and oxygen saturation less than 92% or arterial oxygen partial pressure (PaO_2_) less than 60 mmHg on room air. Hypoxemia severity was assessed using the PaO_2_/fraction of inspired oxygen (FiO_2_) ratio [25]. The decision to perform fiberoptic bronchoscopy and bronchoalveolar lavage (FO-BAL) and the use of life-sustaining treatments (i.e., noninvasive or invasive mechanical ventilation, renal replacement therapy, and vasopressors) was left at the discretion of the attending physicians. Acute respiratory distress syndrome (ARDS) was defined according to the Berlin definition [26]. Therapeutic regimens were reported including antiviral therapy (molecule, dose, and length of treatment) and the use of steroids. High-dose steroids was defined as >30 mg per day of prednisone [27]. Patients receiving steroids were matched in a 1:6 ratio to a control group of patients within this cohort who did not receive steroids. The four matching criteria were: age, year of ICU admission, SOFA score on day 1, and ARDS criteria according to the Berlin definition.

**Table 1:** Univariate analysis of patient characteristics in intubated and nonintubated subjects

	Overall (*n* = 102)	Nonintubated (*n* = 50)	Intubated^a^ (*n* = 51)	*p*
Demographics				
Age, years	39 (32–51)	35 (29.25–40.5)	46 (36.25–55.75)	0.0005
Male gender	64 (63%)	28 (56%)	36 (70.6%)	0.04
Tobacco smokers	52 (51%)	29 (58%)	23 (45.1%)	0.23
Co-morbidities				
Any comorbidity	91 (89%)	41 (82%)	49 (96%)	0.03
Hypertension	14 (14%)	3 (6%)	11 (21.6%)	0.04
Cardiovascular disease	8 (8%)	3 (6%)	5 (9.8%)	0.72
Diabetes	7 (7%)	3 (6%)	4 (7.8%)	1
Chronic kidney disease	7 (7%)	2 (4%)	5 (9.8%)	0.44
Chronic respiratory insufficiency	6 (6%)	2 (4%)	3 (5.9%)	1
Other^b^	5 (5%)	1 (2%)	3 (5.9%)	0.62
Underlying IS	53 (52%)	18 (36%)	35 (68.6%)	0.001
Solid organ transplantation	11 (11%)	5 (10%)	6 (11.8%)	1
Solid malignancy	7 (7%)	1 (2%)	6 (11.8%)	0.11
Hematological malignancy	15 (15%)	4 (8%)	11 (21.6%)	0.09
Pregnancy	6 (6%)	4 (8%)	2 (3.9%)	0.44
Steroids treatment	22 (22%)	7 (14%)	15 (29.4%)	0.09
Other immunosuppression^c^	6 (6%)	1 (2%)	5 (9.8%)	0.20
Respiratory parameters at ICU admission				
Respiratory rate, breaths/min	30 (24.5–35)	25 (22–30)	31 (28.25–39.75)	0.0006
Oxygen flow, L/min	6 (3–15)	4 (2–8)	15 (9.75–15)	<0.0001
PaO_2_:FiO_2_ ratio, mmHg	150 (80–284)	250 (126.5–331)	90 (72–162)	<0.0001
Alveolar consolidation on chest X-ray	30 (33%)	9 (20%)	21 (46.7%)	0.01
SOFA score at day 1	4 (3–7)	3 (2–4)	7 (5–10)	<0.0001
Time (days) from dyspnea onset to ICU admission	2 (1–3)	2 (1–3)	1 (1–3)	0.34
Empirical antibiotics at ICU	62 (61%)	19 (38%)	42 (84%)	<0.0001
Early-documented bacterial co-infection	20 (20%)	4 (8%)	16 (31%)	0.005
Hospital mortality	24 (24%)	2 (4%)	22 (43%)	<0.0001

Values are shown as *n* (%) or median (25^th^–75^th^ percentiles)

^a^One patient was not included in the mechanical ventilation analysis because of intubation 7 days before the diagnosis of varicella-zoster virus-related community-acquired pneumonia and for another reason (urgent surgery)

^b^Other co-morbidities were: cirrhosis (2, 2%), hepatitis B virus chronic infection (1, 1%), hepatitis C virus chronic infection (1, 1%)

^c^Other causes of immunosuppression were: splenectomy (2, 2%), HIV infection (1, 1%), tumor necrosis factor alpha antagonists (1, 1%), methotrexate (1, 1%), azathioprine (1, 1%)

*ICU* intensive care unit, *IS* immunosuppression, *PaO*_*2*_ *:FiO*_*2*_ ratio of arterial oxygen partial pressure to fractional inspired oxygen, *SOFA* Sequential Organ Failure Assessment

**Table 2:** Characteristics of the pulmonary involvement (*n* = 102)

Variables	
Pulmonary symptoms (*n* = 102)	
Temperature, °C	39.2 (38.7–39.9)
Dyspnea	96 (94%)
Cough	45 (44%)
Hemoptysis	9 (9%)
Chest pain	10 (10%)
Acute respiratory failure at ICU admission	69 (68%)
Chest X-ray at ICU admission (*n* = 97, 95%)	
Normal	1 (1%)
Unilateral alveolar opacities	7 (8%)
Bilateral alveolar opacities	23 (24%)
Unilateral interstitial pattern	1 (1%)
Bilateral interstitial pattern	68 (75%)
Lung CT scan (*n* = 31, 30%)	
Normal	0 (0%)
Ground glass opacities	11 (39%)
Nodules	14 (50%)
Consolidations	14 (50%)
Interlobular septal thickening	2 (8%)
Pleural effusion	8 (29%)
Fiberoptic bronchoscopy (*n* = 35, 35%)^a^	
Inflammatory mucosa	6 (43%)
Vesicular lesions on bronchial mucosa	6 (43%)
Normal	2 (14%)
Bronchoalveolar lavage (*n* = 29, 28%)	
Diffuse intra-alveolar hemorrhage^b^	5 (29%)
Cell count/μL (*n* = 11, 38%)	300,000 (215,000–675,000)
Macrophages, % of total cells	50 (22–65)
Lymphocytes, % of total cells	7.5 (5–20)
Neutrophils, % of total cells	31 (5–63)
Eosinophils, % of total cells	0 (0–2)
Siderophages, % of total cells	0 (0–5)
Virus identified by PCR in the BAL (24 samples)	23 (96%)

Values are shown as *n* (%) or median (25^th^–75^th^ percentiles)

^a^Fiberoptic bronchoscopy macroscopic findings were not available for 21 patients

^b^Macroscopic examination of the fluid was not reported in 12 cases

*BAL* bronchoalveolar lavage, *CT* computed tomography, *ICU* intensive care unit, *PCR* polymerase chain reaction

**Table 3:** ICU management and outcome data

Variables	
VZV-related treatment	
Aciclovir	102 (100%)
Aciclovir dose, mg/8 h	10 (10–10)
Steroids	10 (10%)
Immunoglobulins	1 (1%)
Systemic antibiotics at ICU admission	62 (61%)
Primary source of bacterial co-infection (*n* = 40, 39%)	
Lung	24 (60%)
Bloodstream	8 (20%)
Skin	4 (10%)
Other	4 (10%)
Life-sustaining therapies	
Non-invasive mechanical ventilation	29 (28%)
Invasive mechanical ventilation	52 (51%)
Vasopressors	36 (35%)
Renal replacement therapy	24 (24%)
ARDS criteria according to the Berlin definition (*n* = 42, 41%)	
Mild ARDS	8 (19%)
Moderate ARDS	10 (24%)
Severe ARDS	24 (57%)
Other interventions	
Neuromuscular blockers	26/52 (50%)
Prone positioning	14/52 (28%)
Veno-venous ECMO	7/52 (13%)
Outcome data	
ICU length of stay (days)	8 (4–16.75)
Hospital length of stay (days)	14 (9–33)
ICU mortality	17 (17%)
Hospital mortality	24 (24%)

Values are shown as *n* (%) or median (25^th^–75^th^ percentiles)

*AKI* acute kidney injury, *ARDS* acute respiratory distress syndrome, *ECMO* extra-corporeal lung oxygenation, *ICU* intensive care unit, *VZV* varicella-zoster virus

ICU-acquired infections were recorded. The diagnosis of infection was confirmed if patients met both the following criteria: microbiological identification of a pathogen and administration of related antibiotic treatment.

ICU and hospital length of stays, and vital status at ICU and hospital discharge were obtained in all patients.

### Statistics

Patient characteristics at baseline and during ICU stay were described using medians and interquartile ranges for quantitative variables and counts and percentages for qualitative variables. Characteristics of patients requiring mechanical ventilation during their ICU stay were compared to those of patients without mechanical ventilation using either Wilcoxon rank sum test or Fisher’s exact test. To assess variables independently associated with the requirement for mechanical ventilation, baseline characteristics significantly associated with the requirement for mechanical ventilation were included in a multivariable logistic regression model. Due to the small sample size, a forward stepwise *p* value-based variable selection was performed, and, when several highly correlated variables were associated with the requirement for mechanical ventilation, only one was included in the multivariable analysis based on clinical relevance. Missing covariates were handled using multivariate imputation by chained equations. Baseline and ICU management characteristics were also compared between deceased patients and those discharged alive. Cumulative incidence of death in the ICU and death in hospital were estimated considering discharge alive from ICU/hospital as competing events.

To assess the prognostic effect of steroid therapy in the context of an observational cohort, a matched comparison of patients receiving and not receiving steroids was performed. Patients receiving steroids were matched in a 1:6 ratio to a control group of patients within this cohort who did not receive steroids. The four matching criteria were: age, year of ICU admission, SOFA score on day 1, and ARDS criteria according to the Berlin definition.

## Results

### Clinical characteristics

During the study period, we identified 102 adult patients admitted to the ICU for the management of VZV-CAP. There was a seasonal distribution over the study period, with the highest incidence observed during winter and spring (Additional file 2: Figure S1). The median age was 39 (32–51) years and 53 (52%) patients were immunocompromised. Six (6%) cases of VZV pneumonia occurred in pregnant women (Table 1).

A typical chickenpox skin rash was reported in all but four (96%) patients and occurred 3 (2–5) days before the onset of respiratory symptoms. The median time from onset of respiratory symptoms to ICU admission was 2 (1–3) days. On admission, patients were severely hypoxemic with a PaO_2_/FiO_2_ ratio of 150 (80–284) mmHg and 6 (3–15) L/min oxygen flow. Respiratory symptoms included a cough in 45 (44%) patients, chest pain in 10 (10%) patients, and hemoptysis in 9 (9%) patients. ARF was noted in 69 (68%) patients (Table 2). In addition to skin and respiratory symptoms, 13 (13%) patients had encephalitis. Laboratory findings indicated thrombocytopenia in 81 (79%) patients and hepatitis in 34 (33%) patients. Blood PCR to amplify VZV DNA was always positive when performed (*n* = 15, 14.7%).

### ICU management

**Fig. 1:**
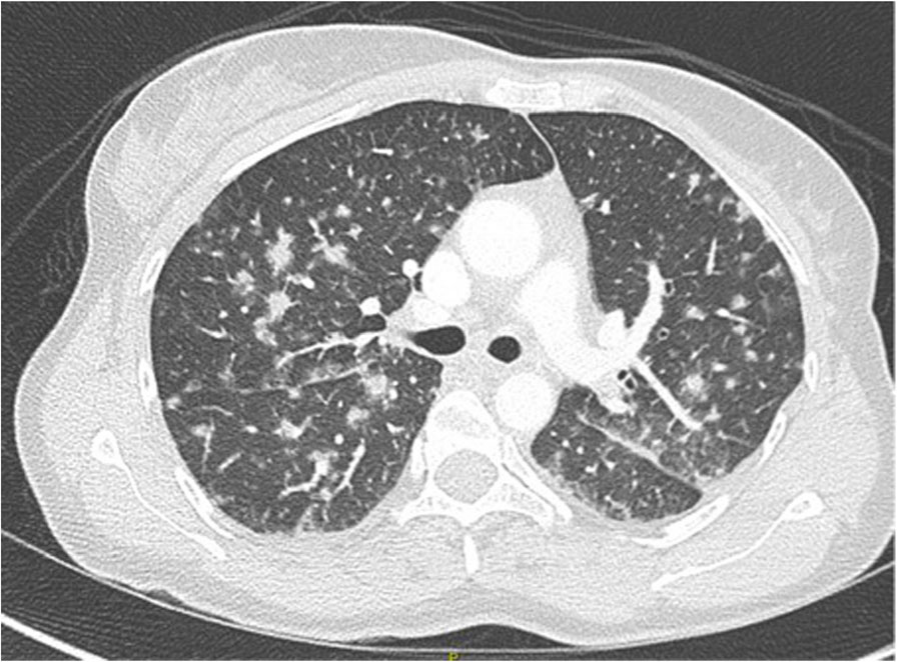
Imaging characteristics from lung CT. A 46-year-old woman was admitted to the ICU for acute respiratory failure. She underwent kidney transplantation 12 years ago. She reported fever and a typical chickenpox skin rash 5 days before admission. The onset of respiratory symptoms started 2 days before ICU admission and invasive mechanical ventilation was implemented at day 1. She developed a severe ARDS requiring prone positioning, neuromuscular blockers, and 14 days of invasive mechanical ventilation. Lung CT scan demonstrated diffuse bilateral nodules, patchy ground glass opacities, and interlobular septal thickening. A fiber bronchoscopy with bronchoalveolar lavage documented a *Staphylococcus aureus* co-infection. She received intravenous aciclovir 10 mg/kg/8 h during 15 days associated with 10 days of oxacilline and was discharge alive from the ICU 17 days after admission

**Fig. 2:**
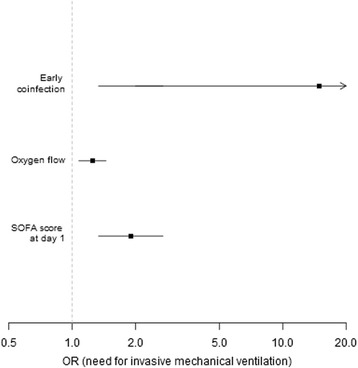
Risk factors associated with the need for invasive mechanical ventilation in patients with VZV pneumonia. *OR* odds ratio, *SOFA* Sequential Organ Failure Assessment

The median SOFA score on day 1 was 4 (3–7). A chest CT was performed in 31 (30%) patients and was never normal. Common abnormalities were diffuse bilateral centrolobular nodules with tree-in-bud appearance (*n* = 14, 50%) and diffuse ground glass opacities (*n* = 11, 39%) (Fig. 1). Alveolar consolidations were also reported (*n* = 14, 50%). Fiberoptic bronchoscopy was performed in 35 (35%) patients and demonstrated vesicular lesions or ulcerations on bronchial mucosa in 13 (36%) cases. PCR for VZV in the bronchoalveolar lavage was tested in 24 patients and yielded a positive result in 96% of cases. Noninvasive mechanical ventilation was implemented in 29 (28%) patients, failing in 19 (66%) who were subsequently intubated. Invasive mechanical ventilation was used in 52 (51%) patients overall, of whom 42 (80.8%) fulfilled the ARDS criteria according to the Berlin definition (Table 3). Patients were intubated 1 (1–2) day after ICU admission. Three factors were independently associated with the need for invasive mechanical ventilation: SOFA score on day 1 (odds ratio (OR) 1.90 (1.33–2.70); *p* < 0.001), oxygen flow at ICU admission (OR 1.25 (1.08–1.45); *p* = 0.004), and early bacterial co-infection (OR 14.94 (2.00–111.8); *p* = 0.009) (Table 4 and Fig. 2). Vasopressors were required in 36 (35%) patients and renal replacement therapy in 24 (24%). Among the 102 patients, 40 (39%) patients had documented bacterial co-infection with 20 (50%) early infections (documented within 72 h after ICU admission) and 20 (50%) late infections. The primary sources of co-infections were the lungs (*n* = 24, 60%), bloodstream (*n* = 8, 20%), and skin (n = 4, 10%), with *Staphylococcus aureus* being the most often recovered pathogen (*n* = 12, 30%). The median ICU and hospital length of stay were 8 (4–16.75) days and 14 (9–33) days, respectively. Duration of mechanical ventilation was 14 (7–21) days.

**Table 4:** Multivariate analysis of factors associated with the need for invasive mechanical ventilation during ICU stay

Variable	OR (CI 95%)	*p*
SOFA score at day 1 (per point increment)	1.90 (1.33–2.70)	<0.001
Oxygen flow at ICU admission, L/min (per L/min increment)	1.25 (1.08–1.45)	0.004
Early bacterial co-infection	14.94 (2.00–111.8)	0.009

Results are presented for the imputed data

Candidate predictors were: age, any comorbidity, underlying immunosuppression, SOFA score at day 1, oxygen flow at ICU admission, alveolar consolidation on chest X-ray, antibiotics at ICU admission, and early bacterial co-infection

*CI* confidence interval, *ICU* intensive care unit, *OR* odds ratio, *SOFA* Sequential Organ Failure Assessment

### Treatments related to VZV pneumonia and outcome

All patients were treated with aciclovir 10 mg/kg/8 h during 11 (9.2–14) days. One (1%) patient received a treatment with a varicella-zoster immune globulin preparation. High-dose adjunctive steroid therapy, in addition to antiviral therapy, was reported in 10 patients. Patients treated with high-dose steroids were no different compared to steroid-free patients regarding demographics, respiratory parameters on ICU admission, and ICU management (Table 5). Compared to steroid-free patients, steroid treatment was associated with a longer duration of mechanical ventilation, and prolonged ICU and hospital stays. ICU mortality was similar in the two groups (20% vs. 20%; *p* = 1.00). ICU-acquired bacterial infections were more frequently reported in steroid-treated patients (80% vs. 43%; *p* = 0.04) (Table 5).

**Table 5:** Characteristics of patients who received steroids (*n* = 10) compared to matched controlled patients (*n* = 60) who did not received steroids^a^

	No steroid use (*n* = 60)	Steroids use (*n* = 10)	*p*
Demographics			
Age, years	43 (34–56)	48 (35.5–60.75)	0.60
Male gender	38 (63%)	5 (50%)	0.49
Co-morbidities	55 (92%)	9 (90%)	1.00
Underlying immunosuppression	33 (55%)	7 (70%)	0.50
Year of ICU admission	2009 (2006–2012)	2010 (2008–2012)	0.48
Time (days) from dyspnea onset to ICU admission	2 (1–3)	2 (1–3.25)	0.79
Parameters at ICU admission			
Temperature, °C	39.1 (38.25–39.7)	39.4 (38.85–40.8)	0.22
Respiratory rate, breaths/min	30 (26.5–38.5)	35 (32–35)	0.34
Oxygen flow, L/min	10 (5–15)	8.5 (1.5–15)	0.55
Hemoptysis	6 (10%)	0 (0%)	0.58
PaO_2_:FiO_2_ ratio, mmHg	105 (75.5–174.5)	86 (80.5–210)	1.00
SOFA score at day 1	5 (4–9)	7 (5–7.75)	0.55
ICU management			
Invasive mechanical ventilation	36 (60%)	9 (90%)	0.08
Time (days) from ICU admission to intubation	1 (1–2)	1 (1–1)	0.30
ARDS criteria according to the Berlin definition	31 (52%)	7 (70%)	0.33
Length of invasive mechanical ventilation, days	13.5 (7–17.25)	28 (13–53)	0.06
Bacterial superinfection	26 (43%)	8 (80%)	0.04
Outcome data			
ICU length of stay, days	10 (4–17)	32 (10.75–69.5)	0.01
Hospital length of stay, days	16.5 (10–32.25)	40.5 (21.25–74)	0.02
ICU mortality	12 (20%)	2 (20%)	1.00
Hospital mortality	15 (25%)	6 (60%)	0.06

Values are shown as *n* (%) or median (25^th^–75^th^ percentiles)

^a^Patients who received steroids were matched in a 1:6 ratio to a control group of patients who did not receive steroids. Matched controls were screened from the current cohort with the following four matching criteria: age, year of ICU admission, SOFA score at day 1, and ARDS criteria according to the Berlin definition

*ARDS* acute respiratory distress syndrome, *ICU* intensive care unit, *PaO*_*2*_ *:FiO*_*2*_ ratio of arterial oxygen partial pressure to fractional inspired oxygen, *SOFA* Sequential Organ Failure Assessment

Overall ICU and hospital mortality were 17% and 24%, respectively, without significant variation over the study period (Additional file 3: Table S3). The main causes of death were: multi-organ failure in 10 patients (42%), refractory ARDS in 5 patients (21%), and septic shock in 4 patients (17%). One (4%) patient died from fulminant hepatic failure attributed to VZV infection and 1 (4%) patient died from brain edema. The cause of death was missing for 3 patients. By univariate analysis, factors associated with hospital mortality were: age (60 (49–72.5) vs. 36 (31–45); *p* < 0.0001), underlying immunosuppression (92% vs. 40%; *p* < 0.0001), SOFA score on day 1 (7 (5–10.75) vs. 4 (2–5.25); *p* = 0.0002), disseminated intravascular coagulation (42% vs. 4%; *p* = 0.0001), and empiric antibiotic treatment on ICU admission (91% vs. 53%; *p* < 0.0001) (Additional file 4: Table S2, Additional file 5: Figure S2, and Additional file 6: Figure S3).

## Discussion

We identified 102 patients with severe forms of VZV-CAP who were admitted to 29 French ICUs. Underlying immunosuppression accounted for half of the patients we evaluated, mainly related to impaired cellular immune response (lymphoproliferative disorders, immunosuppressive drugs for solid-organ transplant recipients, and/or steroid exposure). Nevertheless, we identified severe illness from VZV-CAP among 11 (11%) young and healthy patients. More than half of the patients required invasive mechanical ventilation, of which 80% met ARDS criteria. Risk factors for intubation were related to the severity of the respiratory failure, the presence of an early bacterial co-infection, and the onset of other organ failures. In addition to antiviral therapy, high doses of steroids were used in 10 (10%) patients without benefit either on improvement of respiratory parameters or on mortality, and were associated with an increased number of superinfections.

There was a 5 (3–7)-day period of illness prior ICU admission, which is similar to influenza virus infection and other causes of viral pneumonia [5, 28, 29]. All patients but four presented with a typical chickenpox skin rash, suggesting that VZV-CAP is mostly a dreaded complication of primary VZV infection rather than recurrent VZV infection. The four patients without rash were all immunocompromised (solid cancer or hematological malignancy). Atypical cases of recurrent VZV infection with pulmonary involvement have been reported, almost exclusively in immunocompromised patients, and cannot be excluded in our study [30–33]. In addition, exogenous clinical reinfection by VZV has also been demonstrated and is thought to occur more likely in immunocompromised patients [30]. The illness course was characterized by a short period of acute and severe respiratory deterioration, requiring invasive mechanical ventilation in half of the cases, shortly after ICU admission. We identified that risk factors for intubation were related to the presence of a bacterial co-infection, the severity of the respiratory failure, and the onset of other organ failures. Neither comorbidities nor underlying immunosuppression were independent predictors of invasive mechanical ventilation. These results are in agreement with previous studies suggesting that the underlying medical context was no longer significantly associated with the risk for intubation after adjustment for the severity of the acute disease [34–36]. The factors we identified are easy to assess at the bedside and may contribute to recognizing hospital admission patients who may benefit from early ICU admission.

In our study, the overall mortality was 24% and reached 43% in patients who received invasive mechanical ventilation. Viruses have been increasingly recognized as pathogens responsible for both severe community-acquired and healthcare-associated pneumonia [11, 29]. Choi et al. recently demonstrated in a prospective study that, in the ICU setting, the mortality rate of patients with viral pneumonia was similar to that of patients with bacterial pneumonia [11]. Nevertheless, most of our knowledge on severe forms of viral pneumonia is related to the influenza virus. We report that patients with VZV-CAP who required intubation experienced a long period of invasive mechanical ventilation (14 (7–21) days) and a high rate of bacterial co-infections (69%). These findings might be explained by the extent of skin and mucosal vesicular lesions. Indeed, previous clinical and autopsy studies have demonstrated that these lesions are responsible for necrotic and hemorrhagic foci distributed both in the upper airways and in the lower respiratory tract that may promote bacterial co-infection [37, 38]. In our study, 36% of the patients who underwent fiberoptic bronchoscopy had vesicular lesions on bronchial mucosa.

In addition to antiviral therapy, the use of steroids has been reported by observational studies in the treatment of VZV pneumonia [39–41]. The role of steroids in the management of pneumonia remains controversial. On the one hand, steroids might have the potential to decrease intrapulmonary inflammation and thus to reduce some ARDS-related pulmonary lesions. But, on the other hand, steroids might increase immunosuppression, favor persistent viral replication, and promote superinfection. Based on the results of two randomized controlled trials, the benefit-to-risk ratio argues for its use in severe *Pneumocystis jirovecii* pneumonia in the acquired immunodeficiency syndrome [42, 43]. A recent meta-analysis reported that it may decrease the risk of ARDS as adjunctive therapy in community-acquired pneumonia [44]. On the other hand, no benefit has been demonstrated with the use of steroids in influenza pneumonia [45]. In 1998, Mer and Richards [39] reported on 15 adult patients treated for VZV-CAP, six of whom received steroids in addition to antiviral therapy and supportive care. These six patients experienced clinical improvement, a significantly shorter ICU and hospital length of stay, and no mortality when compared to those who did not receive steroids. However, the study design precluded any robust conclusion. Another study from Saudi Arabia reported improvement in oxygenation parameters in 10 adult patients treated for VZV-CAP with steroids as adjunctive therapy [40]. This was not our experience in the present study. Ten patients received steroids 4.5 (1–15) days after ICU admission. We compared these 10 patients to 60 matched controls who did not receive steroids. There was no difference between the steroid group and the nonsteroid group in ICU and hospital mortality. However, patients treated with steroids had a longer duration of invasive mechanical ventilation, more bacterial superinfection, and an increased ICU and hospital length of stay than control patients not treated with steroids. Thus, our data did not report any benefit associated with the use of steroids as adjunctive therapy in severe VZV-CAP.

Our study has several limitations. First, given the retrospective design over a long period of time, supportive care practices may have changed throughout the study period and influenced the results. However, we report the largest study on the severe forms of VZV-CAP and we believe that it adds valuable data to the current knowledge. Second, we had no biological identification of VZV for all patients included our study. Nevertheless, clinical signs and symptoms of chickenpox are obvious in most cases and laboratory diagnosis is restricted to unusual cases. Thus, we can reasonably assume that the patients included in the present study had VZV-CAP. Third, due to the limited number of death we were unable to identify independent predictors of hospital mortality. However, we report predictors of invasive mechanical ventilation that conceivably may be related to mortality. Fourth, for the diagnosis of VZV-CAP we used a database encoded by physicians at patient ICU discharge and we cannot exclude that some patients with VZV-CAP had been missed.

## Conclusions

In conclusion, severe VZV-CAP in adults is an acute respiratory disease that requires invasive mechanical ventilation in more than half of the cases. Although underlying medical conditions or immunosuppression are common, healthy young individuals may be involved. We identified that respiratory disease severity, early bacterial co-infection, and other organ failures on ICU admission were independent risk factors for invasive mechanical ventilation. Early recognition of these factors may help to identify patients that may benefit from close monitoring to ensure that no treatment delay occurs when intubation is required. Adjunctive steroid therapy did not influence mortality and increased the risk of superinfection.

## Additional files

Additional file 1: Table S1. Participating centers (*n* = 29) with the number of cases of VZV pneumonia during the study period (1996–2016). (DOC 45 kb)

Additional file 2: Figure S1. Seasonal distribution of VZV pneumonia from 1996 to 2015. (TIFF 731 kb)

Additional file 3: Table S3. Mortality evolution during the study period. (DOC 28 kb)

Additional file 4: Table S2. Univariate analysis of patient characteristics associated with hospital mortality. (DOC 39 kb)

Additional file 5: Figure S2. Influence of age on mortality in patients with VZV pneumonia. (TIFF 675 kb)

Additional file 6: Figure S3. Influence of underlying immunosuppression on mortality in patients with VZV pneumonia. (PDF 1968 kb)

## Abbreviations

ARDS: Acute respiratory distress syndrome
ARF: Acute respiratory failure
CAP: Community-acquired pneumonia
CT: Computed tomography
FiO_2_: Fraction of inspired oxygen
FO-BAL: Fiberoptic bronchoscopy and bronchoalveolar lavage
ICD-10: International Classification of Diseases
ICU: Intensive care unit
OR: Odds ratio
PaO_2_: Arterial oxygen partial pressure
PCR: Polymerase chain reaction
SARS: Severe acute respiratory syndrome
SOFA: Sequential Organ Failure Assessment
VZV: Varicella-zoster virus
