# Supplementary material for: Severe varicella-zoster virus pneumonia: a multicenter cohort study
Source: Crit Care. 2017 Jun 7;21:137. doi: 10.1186/s13054-017-1731-0 (PMC5463395; doi:10.1186/s13054-017-1731-0)
Supplement: Supplementary file 1 — Participating centers (n = 29) with the number of cases of VZV pneumonia during the study period (1996–2016). (DOC 45 kb) [file 13054_2017_1731_MOESM1_ESM.doc]

# **Table S1: Participating centers (n=29) with the number of cases of VZV-pneumonia during the study period (1996-2016)**

| **Hospital** | **City** | **Cases of VZV-pneumonia during the study period**  **n (%)** |
| --- | --- | --- |
| Saint-Louis University Hospital | Paris | 5 (5) |
| Limoges University Hospital | Limoges | 8 (8) |
| Poitiers University Hospital | Poitiers | 7 (7) |
| Marseille University Hospital | Marseille | 7 (7) |
| Cochin University Hospital | Paris | 6 (6) |
| René Dubos Hospital | Pontoise | 6 (6) |
| Nantes University Hospital | Nantes | 5 (5) |
| Croix-Rousse University Hospital | Lyon | 5 (5) |
| Saint-Antoine University Hospital | Paris | 4 (4) |
| Hôtel-Dieu Cochin University Hospital | Paris | 4 (4) |
| Henri-Mondor University Hospital | Créteil | 4 (4) |
| Tenon University Hospital | Paris | 4 (4) |
| Bichat University Hospital | Paris | 4 (4) |
| Louis-Mourrier University Hospital | Colombes | 3 (3) |
| Caen University Hospital | Caen | 3 (3) |
| Gabriel-Montpied University Hospital | Clermont-Ferrand | 3 (3) |
| Roger Salengro University Hospital | Lille | 3 (3) |
| Toulouse University Hospital | Toulouse | 3 (3) |
| Pitié-Salpêtrière University Hospital | Paris | 2 (2) |
| Hautepierre University Hospital | Strasbourg | 2 (2) |
| Institut Gustave-Roussy Hospital | Villejuif | 2 (2) |
| Brabois University Hospital | Nancy | 2 (2) |
| Amiens University Hospital | Amiens | 2 (2) |
| Angoulême Hospital | Angoulême | 2 (2) |
| Emile Muller Hospital | Mulhouse | 2 (2) |
| Edouard Herriot University Hospital | Lyon | 1 (1) |
| Rouen University Hospital | Rouen | 1 (1) |
| La Source Hospital | Orléans | 1 (1) |
| Sud-Francilien Hospital | Corbeil-Essonnes | 1 (1) |
| **Total n (%)** |  | **102 (100)** |
