# Supplementary figures and images for: Severe varicella-zoster virus pneumonia: a multicenter cohort study
Source: Crit Care. 2017 Jun 7;21:137. doi: 10.1186/s13054-017-1731-0 (PMC5463395; doi:10.1186/s13054-017-1731-0)

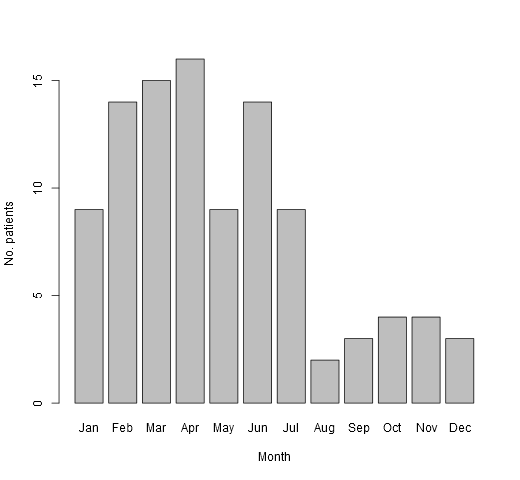

Supplement: Supplementary file 2 — Seasonal distribution of VZV pneumonia from 1996 to 2015. (TIFF 731 kb) [file 13054_2017_1731_MOESM2_ESM.tiff]

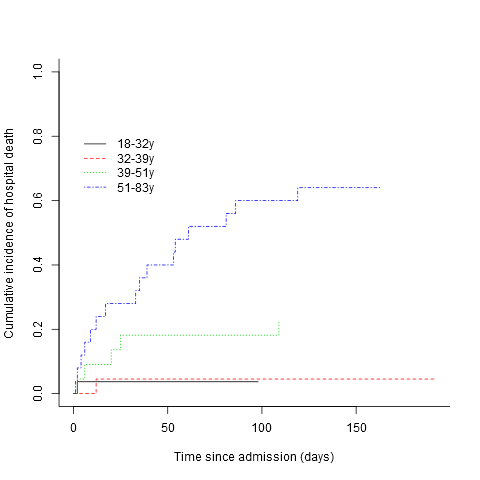

Supplement: Supplementary file 5 — Influence of age on mortality in patients with VZV pneumonia. (TIFF 675 kb) [file 13054_2017_1731_MOESM5_ESM.tiff]

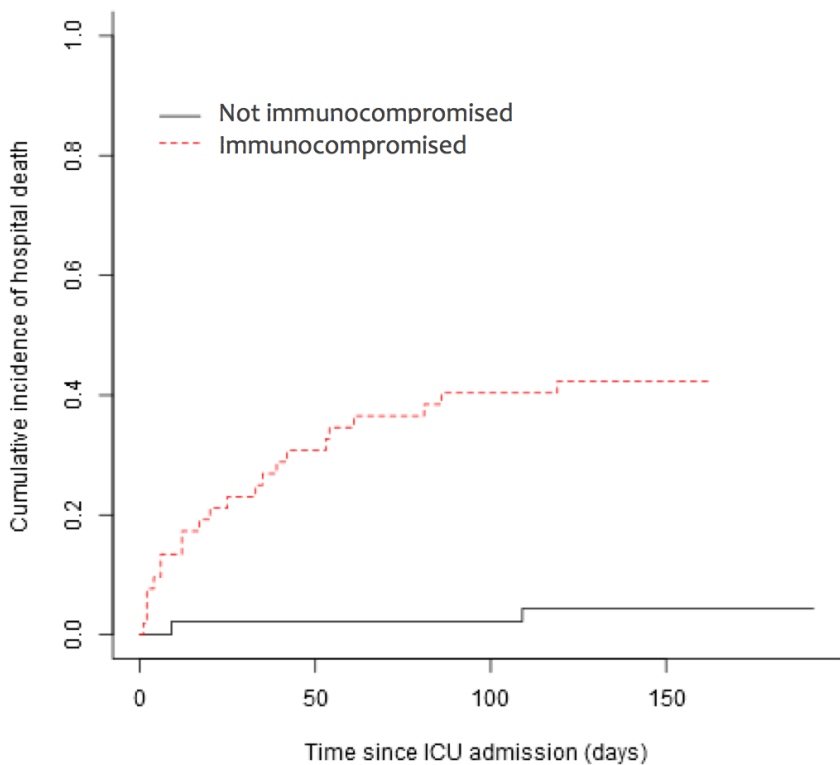

Supplement: Supplementary file 6 — Influence of underlying immunosuppression on mortality in patients with VZV pneumonia. (PDF 1968 kb) [file 13054_2017_1731_MOESM6_ESM.pdf]
