# Supplementary material for: Severe varicella-zoster virus pneumonia: a multicenter cohort study
Source: Crit Care. 2017 Jun 7;21:137. doi: 10.1186/s13054-017-1731-0 (PMC5463395; doi:10.1186/s13054-017-1731-0)
Supplement: Supplementary file 3 — Mortality evolution during the study period. (DOC 28 kb) [file 13054_2017_1731_MOESM3_ESM.doc]

**Table S3: Mortality evolution during the study period:**

| **ICU admission year** | **Overall (n=102)** | **Hospital mortality** | **p** |
| --- | --- | --- | --- |
| **1996-2001** | 10 (10%) | 1 (10%) | 0.19 |
| **2001-2007** | 41 (40%) | 7 (17%) |
| **2007-2014** | 51 (50%) | 16 (31%) |
