# Supplementary material for: Severe varicella-zoster virus pneumonia: a multicenter cohort study
Source: Crit Care. 2017 Jun 7;21:137. doi: 10.1186/s13054-017-1731-0 (PMC5463395; doi:10.1186/s13054-017-1731-0)
Supplement: Supplementary file 4 — Univariate analysis of patient characteristics associated with hospital mortality. (DOC 39 kb) [file 13054_2017_1731_MOESM4_ESM.doc]

# **Table S2: Univariate analysis of patient characteristics associated with hospital mortality.**

| N (%)  or Median (25th-75th percentiles) | Dead at hospital discharge  (n=24) | Alive at hospital discharge  (n = 78) | *p* |
| --- | --- | --- | --- |
| Demographics |  |  |  |
| Age (y) | 60 [49-72.5] | 36 [31-45] | <0.0001 |
| Male gender | 14 (58%) | 50 (64%) | 0.64 |
| Co-morbidities | 24 (100%) | 67 (86%) | 0.06 |
| Year of ICU admission |  |  | 0.19 |
| [1996-2001] | 1 (4%) | 9 (12%) |  |
| [2001-2007] | 7 (29%) | 34 (44%) |  |
| [2007-2014] | 16 (67%) | 34 (44%) |  |
| Underlying immunosuppression | 22 (92%) | 31 (40%) | <0.0001 |
| Respiratory parameters at ICU admission |  |  |  |
| Hemoptysis | 3 (12%) | 6 (8%) | 0.44 |
| Respiratory rate, /min | 30 [26-34.25] | 29 [23-35] | 0.62 |
| Oxygen flow, L/min | 15 [7.5-15] | 6 [2-15] | 0.10 |
| Laboratory parameters at ICU admission |  |  |  |
| DIC | 8 (42%) | 3 (4%) | 0.0001 |
| Chest X-ray at ICU admission (n=97, 95%) |  |  |  |
| Alveolar consolidation on chest X-ray | 9 (47%) | 21 (29%) | 0.17 |
| SOFA score at day 1 | 7 [5-10.75] | 4 [2-5.25] | 0.0002 |
| Time (days) from dyspnea onset to antiviral therapy | 2 [1-3] | 1.5 [1-2.75] | 0.69 |
| Antibiotics at ICU admission | 21 (91%) | 41 (53%) | <0.0001 |

Definition of abbreviations: DIC = Diffuse Intravascular coagulation; ICU = Intensive Care Unit; SOFA = Sequential Organ Failure Assessment.
